# The Interplay between Dyslipidemia and Neighboring Developments in Coronary Artery Disease Progression: A Personalized Approach

**DOI:** 10.3390/jpm14030237

**Published:** 2024-02-23

**Authors:** Tomasz Urbanowicz, Krzysztof Skotak, Anna Olasińska-Wiśniewska, Krzysztof J. Filipiak, Jakub Bratkowski, Beata Krasińska, Zbigniew Krasiński, Andrzej Tykarski, Marek Jemielity

**Affiliations:** 1 Cardiac Surgery and Transplantology Department, Poznan University of Medical Sciences, 61-701 Poznan, Poland; 2 Institute of Environmental Protection–National Research Institute, 02-170 Warsaw, Poland; 3 Institute of Clinical Science, Maria Sklodowska-Curie Medical Academy, 00-136 Warsaw, Poland; 4 Department of Hypertensiology, Angiology and Internal Medicine, Poznan University of Medical Sciences, 61-701 Poznan, Poland; 5 Department of Vascular, Endovascular Surgery, Angiology and Phlebology Medical University, Poznan University of Medical Science, 61-701 Poznan, Poland

**Keywords:** coronary disease, chronic coronary syndrome, arterial hypertension, dyslipidemia, nontraditional risk factors, Gensini, familiar development

## Abstract

(1) Background: Estimates suggest that up to 10% of global annual cardiovascular deaths could be related to environmental factors. Not only air pollution components, but also noise exposure and climate changes, are highlighted as nontraditional causes of cardiovascular morbidity. The aim of this study was to identify possible urbanization risk factors for the progression of coronary artery disease in a group of patients with chronic coronary syndrome. (2) Method: There were 77 patients (50 (65%) males and 27 (35%) females) with a median age of 70 (60–74) years who underwent repetitive angiography due to chronic coronary syndrome between 2018 and 2022. The Gensini score was calculated for assessment of coronary artery disease advancement. Environmental factors including neighboring developments were taken into account in this analysis, including housing, commercial, and industrial developments within 300, 500, and 700 m distances (buffer) from the place of habitation. (3) Results: The multivariable analysis results for prediction of Gensini score progression in relation to 700 m buffer urbanization pointed out the significance of hyperlipidemia (OR: 4.24, 95% CI 1.34–13.39, *p* = 0.014), initial Gensini score (OR: 1.02, 95% CI 1.00–1.05, *p* = 0.112), and neighborhood housing (OR: 0.03, 95% CI 0.01–0.49, *p* = 0.025). (4) Conclusions: Hyperlipidemia and housing neighborhood can be regarded as possible factors for coronary disease progression in patients with chronic coronary syndrome with the use of optimal medical therapy.

## 1. Introduction

In addition to traditional risk factors for coronary artery disease, the prognostic value of environmental elements for health and particularly the cardiovascular system has gained much attention in recent years [1,2]. Projections indicate that up to 10% of annual global deaths can be attributed to noncommunicable disorders, such as chronic respiratory, cardiovascular, and metabolic diseases [3]. Both industrial and non-human-related disasters may lead to significant changes in air pollution and a substantial increase in population mortality [4,5,6]. Observational studies indicate environmental factors as an origin of atherosclerosis in up to 25% of cases [7]. Individual exposure to air pollution in combination with external environmental risk factors is underlined as detrimental [8]. The impact of the types and quality of development on occupants’ condition has been reported [9], though there are substantial gaps in the evidence.

The significant differences in atherosclerosis progression risk related to urban and rural regions have already been narrated [10,11]. Not only air pollution components [12] but also noise exposure [13] and climate changes [14] are highlighted as causative features for cardiovascular morbidity. The possible mediatory role of green spaces on traditional risk factors for atherosclerosis such as dyslipidemia and arterial hypertension was determined [15].

The relation between urbanization described by building types in the habitation area and cardiovascular risk is still under investigation. Housing areas can be defined by higher green space density and lower car traffic intensity. Industrial development is characterized by higher air pollutant fabrication, while commercial development can be described by increased noise and car-related ambient air pollutants.

The air pollution and environmental changes due to industrial expansion are currently treated as the most important public health problems. Several attempts have been undertaken in public health services to improve controlling air pollution and reduce health risks to acceptable levels and standards [16].

The aim of this study was to identify possible urbanization risk factors for coronary artery disease progression in a group of patients with chronic coronary syndrome despite optimal medical therapy. Environmental factors in terms of neighboring developments were taken into analysis, including housing, commercial, and industrial developments within 300, 500 and 700 m distances (buffer) from the place of habitation.

## 2. Materials and Methods

There were 76 patients (50 (66%) males and 26 (34%) females) with a median age of 70 (60–74) years who underwent repetitive coronary angiography due to chronic coronary syndrome between 2018 and 2022. Detailed demographic and clinical histories were collected. The group was characterized by coexisting arterial hypertension (44 (58%)), dyslipidemia (42 (55%)), diabetes mellitus (19 (25%)), and chronic obstructive pulmonary disease (COPD) (4 (5%)).

All the patients were treated according to contemporary guidelines on the treatment of chronic coronary syndromes, including with antiplatelets, angiotensin-converting enzyme inhibitors or angiotensin receptor blockers, beta-blockers, and statins or a statin/ezetimibe combination.

All patients were treated according to current guidelines, including with B-blockers, ACE-I, and statin therapy aimed at lowering the LDL cholesterol fraction below the thresholds recommended according to the patient’s individual cardiovascular risk profile. In patients who underwent percutaneous coronary intervention (PCI), double antiplatelet therapy was continued for up to 6 months depending on the operators’ recommendation.

This retrospective observational descriptive single-center analysis was conducted in accordance with the Declaration of Helsinki and approved by the Institutional Review Board (or Ethics Committee) of Poznan University of Medical Sciences, Poznan, Poland (protocol code 969/23 from 6 December 2023) for studies involving humans.

### 2.1. Dependent Variables

Patients were divided into two groups according to their coronary artery disease progression in repeated coronary angiographies performed at intervals of 381 (148–875) days. Coronary atherosclerotic lesions described by the Gensini score [17] were analyzed. Identification of progression was based on the development of new lesions in coronary arteries based on changes in the Gensini score. Group 1 (no progression) served as a control for Group 2 (progression).

### 2.2. Urbanization Assessment Methodology

For identification of urbanization factors corresponding to land use in patients’ places of habitation, The Database of Topographic Objects (BDOT10k) was used.

The Database of Topographic Objects in Poland, containing objects such as official topographic maps, has been elaborated on in digital technologies since the 1990s based on the legal basis of the Geodetic and Cartographic Act of Law [18]. The basic task of BDOT10k creation is to collect and share data for use in various information systems created by public and private institutions based on common organization technical standards, including format procedures (incl. the UML application scheme and GML scheme). All data are gathered and processed based on an object catalog with three-level classification [19]. The urbanization database was created and is updated on the basis of data from various sources, incl. land and building registers, the state register of geographical names with physiographic objects, a numerical terrain model, and orthophotomap and satellite imaging. BDOT10k is a spatial database of a reference nature corresponding to a detailed topographical map in the scale 1:10,000, and collects information about buildings such as their spatial location, cartographic codes, and building characteristics. All objects, including buildings, are grouped on three levels: on the first there are categories of object classes, on the second there are object classes, and on the third, most detailed one there are objects themselves [20]. The most detailed third level for buildings includes five classes: multi-family buildings, single-family buildings (detached houses), industrial storage buildings, shopping buildings, and service buildings and other unclassified buildings.

The role of urban planning in improving the well-being of city residents plays an increasingly important part in protecting public health, especially in defining spatial construction of residential complexes with not only easy access to public transport, schools, or kindergartens, but mostly for low-density urbanization and green areas. Based on BDOT10k, the shares of space occupied by housing (incl. multi-family and single-family building classes), commercial (shopping and service buildings), and industrial developments (industrial storage buildings) in describing patients’ immediate places of residence were calculated. In our analyses, we included three different areas defined by 300, 500, and 700 m straight-line distances (buffers with surface areas of about, respectively, 28.3 ha, 78.5 ha, and 153.9 ha). Straight-line distance from place of habitation allows residents to access any areas in approximately 10–15 min on foot for the 300 m buffer and about 20 min on foot for a distance of 700 m [21]. The 500 m buffers described common land use characteristics close to the inhabited area.

### 2.3. Statistical Analysis

The normality of the distribution of variables was tested with the Shapiro–Wilk test. The *t*-test, Cochran–Cox test, Mann–Whitney tests, or Fisher’s exact test were used where applicable to compare the variables between the two groups. Logistic regression was performed to analyze the laboratory data, which predicted the progression of coronary artery disease. Spearman correlation analysis was used to describe the correlation between the variables. Statistical analysis was performed using Jasp version 0.14.1 13 by BibTex. *p* < 0.05 was considered statistically significant. Receiver operator characteristic (ROC) analysis was carried out with SPSS version 29 (IBM SPSS Statistics, New York, NY, USA).

## 3. Results

The total study group of 76 patients was divided according to progression of coronary artery disease. There were 32 patients (21 men and 11 women) with a median age of 71 (62–75) years enrolled into Group 1 who were characterized by no Gensini score progression on repeated coronary angiograms.

The analysis was performed between 2018 and 2022 in the Wielkopolska region of Poland and the enrolled groups were characterized by repeated angiography due to stable coronary syndrome within time intervals of 381 (148–875) days.

Group 2 comprised 44 patients (29 men and 15 women) with a median age of 69 (62–75) years who presented coronary atherosclerosis progression defined by a Gensini score increase. There were no significant differences between clinical characteristics or time intervals between coronary angiographies (*p* = 0.490) between the two subgroups, as presented in Table 1.

Normal angiograms were revealed in 10 (31%) and 9 (21%) patients in Groups 1 and 2, respectively (*p* = 0.122). Percutaneous coronary intervention (PCI) was performed in 22 (69%) and 35 (80%) patients, respectively, resulting in nonsignificant Gensini scores after the procedure (*p* = 0.131). The time intervals between initial and repetitive coronary angiography were 383 (242–889) and 371 (124–813) days (*p* = 0.490) in Groups 1 and 2, respectively.

There were no significant differences related to demographic and clinical factors between the two groups. The study characteristics group showed 8% current nicotine addiction. The study was conducted in an Eastern European country among white non-Latino patients, and the majority of them were retired. The lifestyle profiles regarding diet and activity were comparable.

Environmental factors including neighboring developments were taken into account in this analysis, including housing, commercial, and industrial developments within 300, 500, and 700 m straight-line distances from the place of habitation, and are presented in Table 2.

**Figure 1:**
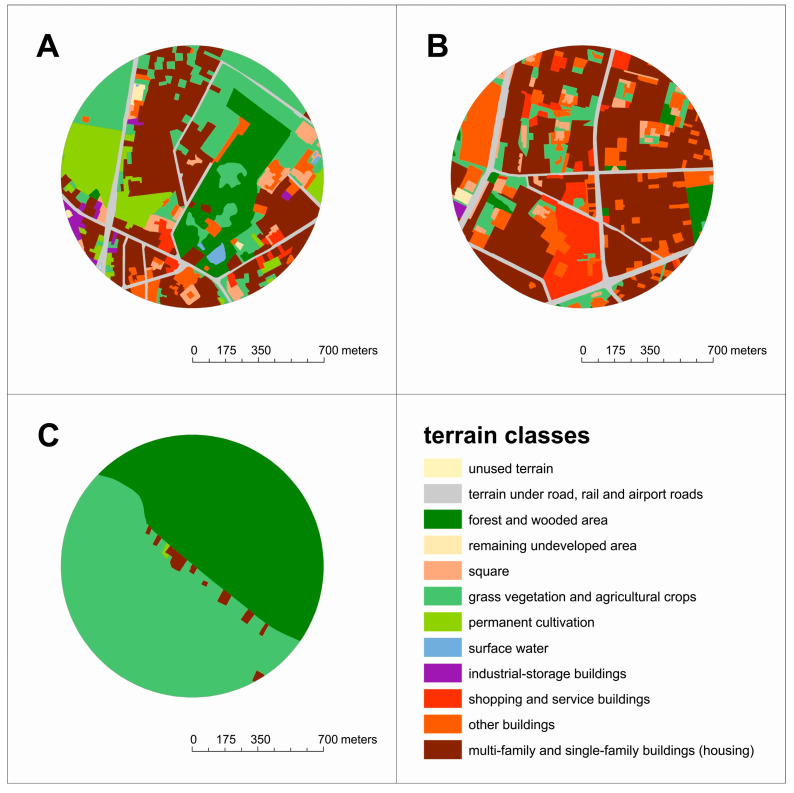
The scope of land use in the patients’ places of living within a 700 m buffer. (**A**) Areas typical for most patients (median), (**B**) urbanized areas (maximum), (**C**) nonurban areas (minimum).

The analysis of land use in the neighborhood of each analyzed patient’s place of habitation (distance up to 700 m) showed significant differences related to characteristics of cities, suburban areas, and rural areas. The lowest share of housing areas was less than 2% (only single-family houses) with a significant share of cultivated land and meadows (51%), as well as forest and wooded areas (47%), as shown in Figure 1c. The places of residence of patients living in highly urbanized zones looked different, where housing areas dominated at the level of 76% (multi- and single-family buildings accounted for 30% and 20%, respectively, commonly with industrial and shopping and service buildings around). In these surroundings, green areas generally did not exceed 12% in total (Figure 1b).

It should be emphasized that a significant number of patients in Poland live in typical areas with buildings at the level of 40% (with 20% single- and 9% multi-family buildings), while approximately 48% of land is surrounded and interspersed with green areas (dominated by lawns—25%, green and blue urban infrastructure—15%, and city and suburban forests—approx. 10%), as shown in Figure 1a.

### 3.1. Logistic Regression

Univariable and multivariable analyses for the prediction of coronary artery disease progression were performed. The significance of dyslipidemia as a predictive factor for Gensini score progression was confirmed. Backward stepwise logistic regression was performed including exposure to neighborhood housing and industrial and commercial development density for 28.3 ha (300 m straight-line distance to the place of habitation), 78.5 ha (500 m buffer), and 153.9 ha (700 m).

The multivariable analysis results for prediction of the Gensini score progression in relation to 300 m urbanization pointed out dyslipidemia as the sole factor (OR: 2.83, 95% CI 1.05–7.66, *p* = 0.040).

The multivariable analysis results for prediction of the Gensini score progression in relation to 500 m urbanization pointed out dyslipidemia (OR: 3.51, 95% CI 1.18–10.41, *p* = 0.024), initial Gensini score (OR: 1.02, 95% CI 0.99–1.05, *p* = 0.152), and neighborhood housing (OR: 0.07, 95% CI 0.01–0.66, *p* = 0.101) as factors, as presented in Table 3.

The multivariable analysis results for Gensini score progression prediction in relation to 700 m buffer urbanization pointed out dyslipidemia (OR: 4.24, 95% CI 1.34–13.39, *p* = 0.014), initial Gensini score (OR: 1.02, 95% CI 1.00–1.05, *p* = 0.112), and neighborhood housing (OR: 0.03, 95% CI 0.01–0.49, *p* = 0.025) as factors.

**Figure 2:**
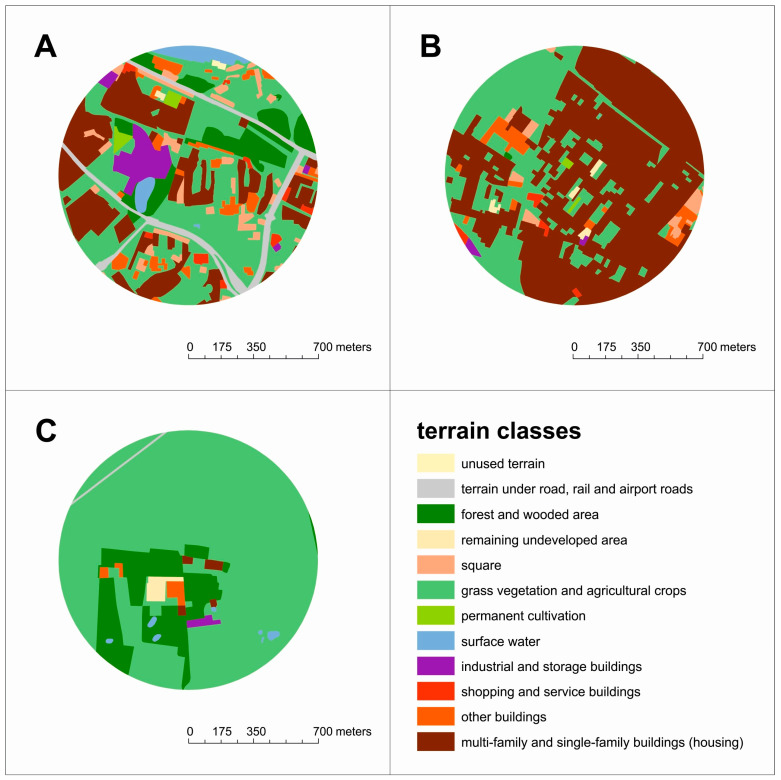
The scope of land use for (**A**) medium, (**B**) maximum, and (**C**) minimum patient results from the multivariable analysis results in relation to 700 m buffer.

The analysis of land use for patients with minimum values in the multivariable analysis in the 700 m buffer shows 2% housing areas (less than 1% single-family houses and 1% other buildings) with domination by cultivated land and meadows (81%) and forests (16%), as shown in Figure 2c. The characteristics of land use in the surroundings of patients with maximum values in the multivariable analysis confirm the dominant role of the presence of 65% housing areas (with 63% single-family buildings and above 1% industrial and shopping/service buildings) and 30% agricultural green areas, as shown in Figure 2b. The greatest diversity in land development is visible for patients with median values in the multivariable analysis, with buildings at the level of 30% (with 21% multi-family buildings, only 3% single-family buildings, and 4% industrial storage buildings) and 41% grasses, as shown in Figure 2a.

### 3.2. Receiver Operator Curve (ROC)

#### 3.2.1. Three Hundred-Meter Buffer (28.3 Ha Area around Place of Habitation)

**Figure 3:**
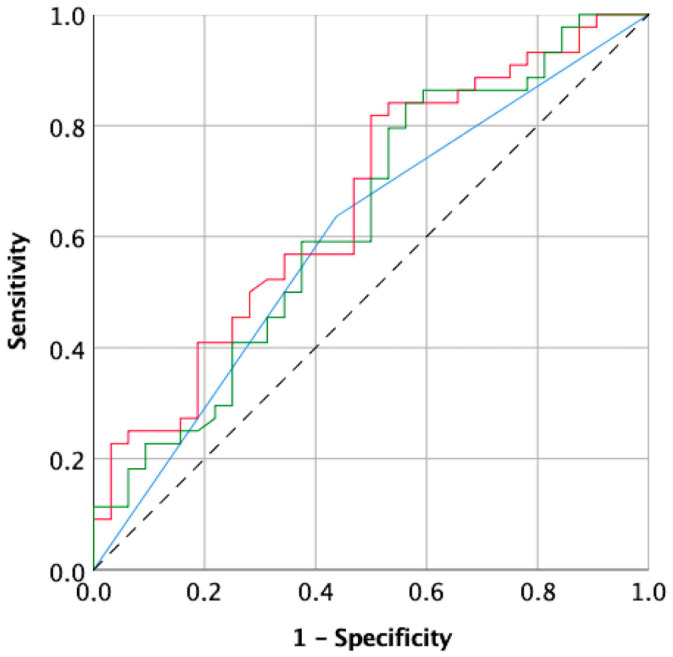
Receiver curve analysis for coronary artery prediction. MODEL 1—blue line—300 m buffer (dyslipidemia), MODEL 2—red line—500 m buffer (dyslipidemia, Gensini score, housing neighborhood), MODEL 3—green line—700 m buffer (dyslipidemia, initial Gensini score, and housing neighborhood).

The multivariate analysis and ROC analysis revealed the predictive value of dyslipidemia for the prediction of atherosclerotic lesion progression estimated by the Gensini score, yielding a sensitivity of 63.6% and specificity of 56.3% and an area under the curve (AUC) of 0.600 (95% CI 0.469–0.729), presented as MODEL 1 in Figure 3.

#### 3.2.2. Five Hundred-Meter Buffer (78.5 Ha Area around Place of Habitation)

The multivariate analysis and ROC analysis revealed the predictive value of dyslipidemia, initial Gensini score, and housing neighborhood for the prediction of atherosclerotic lesion progression estimated by the Gensini score, yielding a sensitivity of 72.7% and specificity of 50.0% and an area under the curve (AUC) of 0.660 (95% CI 0.536–0.784), presented as MODEL 2 in Figure 3.

#### 3.2.3. Seven Hundred-Meter Buffer (153.9 Ha Area around Place of Habitation)

The multivariate analysis and ROC analysis revealed the predictive value of dyslipidemia, initial Gensini score, and housing neighborhood for the prediction of atherosclerotic lesion progression estimated by the Gensini score, yielding a sensitivity of 59.1% and specificity of 46.9% and an area under the curve (AUC) of 0.629 (95% CI 0.500–0.758), presented as MODEL 3 in Figure 3.

## 4. Discussion

The results of our retrospective analysis of possible factors for Gensini score progression in patients with chronic coronary syndrome indicate hypercholesterolemia alone or combined with primary coronary atherosclerosis complexity and neighborhood housing. The latter parameter and its potentially protective role against disease advancement among patients with the use of optimal medical therapy are novel and indicate unique and conceivable directions for future modification.

Our analysis pointed out the optimal results presented by the receiver operator curve analysis for a 500 m buffer (78.5 ha area around the living place). In a previous meta-analysis, urban residential surroundings in addition to exposure to chemicals and heavy metals, noise, air pollution, and ambient temperature were found as significant factors in human morbidity [22].

Currently, many clinical factors can be effectively modified due to the use of promotional and awareness-raising campaigns among patients diagnosed with coronary artery disease. Air pollution, climatic changes, and urbanization are underlined as novel possible factors with significant impact on atherosclerotic lesion progression. Oxidative stress mechanism induction by air pollution has been referred to as causing gene activation and subsequent inflammation, triggering monocyte activation and immune cell adhesion and infiltration, followed by endothelial dysfunction, foam cell formation, and atherosclerotic plaque destabilization [23].

Sun et al. [24] in their study pointed out the association between PM_2.5_, PM_10_, and NO_2_ and increased risk for coronary artery disease progression. In our previous personalized analysis, we found a correlation between coronary disease progression and exposure to NO_2_ in city inhabitants [25]. Rinaldi et al. [26] investigated patients with acute coronary syndrome and noticed the relation between plaque rupture and a steady increase in daily PM_2.5_ exposure levels. Exposure to a short-term increase in PM_2.5_ pollution resulted in emergency department visits for acute coronary syndromes and pneumonia, in contrast to PM_10_, which was associated with a hospitalization upsurge due to acute heart failure and asthma [27].

Among urbanization factors, the observed exposure to excessive noise including from aircraft was claimed to be related to acute coronary syndrome reoccurrence [28]. Authors have stated that environmental noise exposure reduction may provide substantial benefit in improving clinical outcomes. Munzel et al. [29] postulated the multipotential results of noise on human health, including oxidative stress activation, gene network alteration, and harmful effects for the neuronal–cardiovascular axis.

Reduced bioavailability of nitric oxide (NO), secondary to excessive reactive oxygen species (ROS), provokes endothelial dysfunction not only in traditional cardiovascular risk factors such as hypercholesterolemia, diabetes, or hypertension, but also in conditions of environmental stress [30]. The latter factors implicate not only impaired NO signaling, but also an increase in stress hormone levels and inflammatory cytokine concentration [31]. The disruption of endothelium function is claimed as critical for cardiovascular physiology [32].

In our analysis, the multivariable regression followed by the ROC curve analysis pointed out the meaningful character of urbanization in relation to atherosclerosis progression. The influence of urbanization, described as distance-related family housing, commercial development, and industrial development, on coronary disease progression is the primary finding. Distance-related buffer zones are not only justifiable according to our logistic regression results, but also indicate the protective effect of neighborhood buildings on human health, in contrast to commercial and industrial development areas, which are often perceived as less health-friendly due to noise, poorer air quality, and climatic conditions. With a broader living buffer covering a larger surface area that was predominated by housing and with less industrial and commercial development, a more significant environmental impact on coronary disease progression was noticed.

Modulations of the neighborhood-dependent cardiovascular disease risk factors related to socioeconomic status and society-related lifestyle adjustments have already been presented [33]. The numerous nontraditional cardiovascular disease risk factors may interfere with inflammatory activation, resulting in atherosclerotic plaque progression due to various interactions, partially overlapping mechanisms, endocrine signaling, and autophagy [34]. Chronic exposure to environmental stressors, including air pollution, climatic changes, and urbanization characteristics, contributes significantly to noncommunicable disease [35]. It is considered that three quarters of the global burden of cardiovascular diseases in the current population can be prevented, as recent evidence indicates that environmental stressors contribute significantly to premature events [36]. Wilker et al. [37], in their comparative study, revealed higher survival rates after ischemic stroke in patients living in residential proximity to green spaces. In contrast, in Zijlema et al.’s analysis [38], no association was presented between mortality risk and two or more natural spaces being located around the living zone.

The role of urbanization described by household density and altitude was found to be significant for CV risk [39]. Urbanization is asserted as a strong moderator and is believed to be gauged effectively [40]. Our analysis, which is novel in terms of evaluating the relationship between development and atherosclerosis, presents the personalized characterization of urbanization measured by households, commercial developments, and industrial developments in a buffer distance. The novelty of our analysis is based on local environmental characteristics presented by buffer zones that represent the concerned elderly habitants [41]. Ranta et al., in their review [42], presented the interplay between health status, especially stroke risk, and environmental factors such as urbanization, atmospheric factors, chemical contamination, and climate change. Chandy et al. [43], in their experimental study, revealed the pivotal role of gene–environment interactions that may predispose some individuals to adverse cardiovascular outcomes. They investigated induction of pluripotent stem cells (iPSCs) under environmental stressors, indicating genetic polymorphisms resulting in individually related susceptibility to toxins.

Yuan et al. [44], in their meta-analysis, did not reveal an association between cardiovascular mortality and green spaces, while an age-related reduction in morbidity was observed. In opposition to previous studies, our analysis focused on development density in a walking distance buffer from the place of habitation.

There are four categories that link housing and neighborhood conditions with human health, including residential instability, financial burdens, housing safety and quality, and neighborhoods [44]. The source of indoor pollutants related to housing conditions, mainly in degraded buildings, and their impact on human health are postulated [45]. Not only internal housing conditions but also stability and affordability in addition to neighborhood are among the postulated factors. Living areas are characterized by cultural and social values together with physical characteristics. It should be emphasized that the interactivity between human mental and physical health and living areas is complex.

Previous reports have suggested that human morbidity is influenced by such parameters of external buffers as exterior noise sources or insufficient daylight [46].

The buffers created in our analysis were based on walking distance criteria (access to) from the living place and included 300, 500, and 700 m distances. In our results, the 500 m buffer presented the optimal predictive characteristics, while housing neighborhood was combined with clinical factors such as dyslipidemia and coronary artery disease advancement. The buffer zones characterized by household buildings and commercial and industrial development indicate novel and significant urbanization risk factors in our environment. Intensified car traffic with secondary noise and carbon and nitrogen oxide air pollution characterizes commercial zones, and increased ambient particulate matter combined with contained heavy trace metal or/and polycyclic aromatic hydrocarbon contamination very much characterize industrial zones. In previous reports, the 300 m green space buffer was associated with reduced mortality [47]. A relation between 500 m buffer green space and newborn gestational age was presented [48]. Moreover, a relation between type 2 diabetes and extensive traffic exposure was shown by Zhao et al. [49].

In contrast, household buildings are surrounded by green zones, pedestrian paths, and bicycle paths. In our analysis, the protective effect of neighborhood housing may be explained by backyards, gardens, lawns, and playgrounds and the lack of air pollutant emission from multi-family buildings.

### Study Limitations

This study was performed as a retrospective single-center analysis and therefore a limited number of patients was involved since the personalized approach required individual exposure estimation. Urbanization was calculated in cities with no skyscrapers and with a maximum square km population of 2062.

We decided to adjust the study time to a period with fewer possible changes in urbanization. We were aware that longer periods would interfere with our results in terms of changes in modernization and industrial development.

We did not include particulate matters 2.5 and 10 or noise measurements to avoid implementation of too large a number of possible factors, which would diminish the power of the multivariable analysis. We are, however, aware of the value of these air pollution parameters [50].

The assessment of the individual patients’ progression and related cardiovascular and nonclassical risk factors is the main strength of our study.

## 5. Conclusions

An individualized approach to possible CV risk factors is advisable, as it is significantly different in head-to-head comparison regarding nontraditional factors. Apart from dyslipidemia and arterial hypertension, housing neighborhood can be regarded as a possible protective factor against the progression of coronary artery disease in patients with chronic coronary syndrome treated with optimal medical therapy. Patients’ living places being surrounded by familiar development may play an increasing role in the evolution of atherosclerotic disease.

## Figures and Tables

**Table 1:** Patients’ characteristics and angiographic results.

Parameters	Group 1 (No Progression) *n* = 32	Group 2 (Progression) *n* = 44	*p*
Demographic:			
Sex (M (%)/F (%))	21 (66)/11 (34)	29 (66)/15 (34)	0.985
Age (years) (median (Q1–Q3))	71 (62–75)	69 (60–73)	0.188
Weight (kg) (median (Q1–Q3))	76 (69–95)	85 (71–95)	0.632
Height (cm) (median (Q1–Q3))	167 (160–175)	163 (156–174)	0.389
BMI (median (Q1–Q3))	23 (21–28)	25 (20–32)	0.215
Clinical:			
HA (*n* (%))	18 (56)	26 (59)	0.810
Dyslipidemia (*n* (%))	14 (44)	28 (64)	0.088
DM (*n* (%))	7 (22)	12 (27)	0.599
Thyroid (*n* (%))	1 (3)	5 (11)	0.195
Kidney (*n* (%))	3 (9)	4 (9)	0.975
CV disease (*n* (%))	4 (12)	9 (21)	0.490
COPD (*n* (%))	1 (3)	3 (7)	0.488
Coronary angiograms:			
Normal angiograms (*n* (%))	10 (31)	9 (21)	0.122
PCI (*n* (%))	22 (69)	35 (80)	0.290
Gensini 1 after PCI (median (Q1–Q3))	1 (0–5)	5(0–10)	0.131
Gensini 2 (median (Q1–Q3))	1 (0–5)	13.5 (8–27.5)	<0.001
Time interval (days) (median (Q1–Q3))	383 (242–889)	371 (124–813)	0.490

Abbreviations: BMI—body mass index, cm—centimeter, COPD—chronic obstructive pulmonary disease, CV—cardiovascular, HA—arterial hypertension, DM—diabetes mellitus, kg—kilograms, *n*—number, PCI—percutaneous coronary intervention, Q—quartiles.

**Table 2:** The environmental characteristics described by type of building urbanization density.

Parameters	Group 1 *n* = 32	Group 2 *n* = 44	*p*
Urbanization related to distance:			
- 300 m (28.3 hectares)	0.435 (0.291–0.671)	0.467 (0.334–0.626)	0.570
- 500 m (78.5 hectares)	0.394 (0.248–0.552)	0.426 (0.226–0.568)	0.883
- 700 m (153.9 hectares)	0.334 (0.217–0.498)	0.374 (0.224–0.484)	0.846
300 m distance development:			
- Housing	0.344 (0.233–0.471)	0.365 (0.227–0.458)	0.962
- Industrial	0.009 (0.000–0.020)	0.004 (0.000–0.015)	0.257
- Commercial	0.005 (0.000–0.042)	0.012 (0.002–0.034)	0.479
500 m distance development:			
- Housing	0.282 (0.158–0.375)	0.302 (0.189–0.394)	0.854
- Industrial	0.014 (0.003–0.028)	0.009 (0.002–0.021)	0.381
- Commercial	0.011 (0.002–0.028)	0.018 (0.004–0.038)	0.302
700 m distance development:			
- Housing	0.226 (0.150–0.366)	0.246 (0.155–0.332)	0.937
- Industrial	0.023 (0.004–0.045)	0.018 (0.05–0.034)	0.621
- Commercial	0.010 (0.002–0.024)	0.012 (0.001–0.037)	0.304

Abbreviations: m—meter, *n*—number.

**Table 3:** The regression analyses for coronary lesion progression related to demographic and clinical factors and urbanization.

	Univariable			Multivariable		
300 m Distance (28.3 Hectares)						
Parameters	OR	95% CI	*p*	OR	95% CI	*p*
Demographic and clinical factors:						
Sex	1.01	0.39–2.64	0.979	-	-	-
Age	0.97	0.92–1.02	0.203	-	-	-
BMI	1.02	0.99–1.05	0.276	-	-	-
HA	1.75	0.69–4.42	0.236	-	-	-
DM	1.34	0.46–3.90	0.592	-	-	-
Hyperlipidemia	2.25	0.89–5.70	0.087	2.83	1.05–7.66	0.040
CV disease	1.65	0.50–5.47	0.409	-	-	-
Coronary angiography:						
Primary Gensini	1.01	0.99–1.03	0.392	-	-	-
Primary PCI	1.71	0.62–5.03	0.286	-	-	-
Time interval	1.00	0.99–1.00	0.611	-	-	-
Development:						
- Housing	0.67	0.05–1.32	0.752	-	-	-
- Industrial	1.46	1.23–6.23	0.727	-	-	-
- Commercial	2.49	1.45–4.23	0.893	-	-	-
**500 m Distance (78.5 Hectares)**						
Parameters	OR	95% CI	*p*	OR	95% CI	*p*
Demographic and clinical factors:						
Sex	1.01	0.39–2.64	0.979	-	-	-
Age	0.97	0.92–1.02	0.203	-	-	-
BMI	1.02	0.99–1.05	0.276	-	-	-
HA	1.75	0.69–4.42	0.236	-	-	-
DM	1.34	0.46–3.90	0.592	-	-	-
Hyperlipidemia	2.25	0.89–5.70	0.087	3.51	1.18–10.41	0.024
CV disease	1.65	0.50–5.47	0.409	-	-	-
Coronary angiography:						
Primary Gensini	1.01	0.99–1.03	0.392	1.02	0.99–1.05	0.152
Primary PCI	1.71	0.62–5.03	0.286	-	-	-
Time interval	1.00	0.99–1.00	0.611	-	-	-
Development:						
- Housing	0.50	0.06–1.47	0.531	0.07	0.01–0.66	0.101
- Industrial	1.78	1.34–7.49	0.459	-	-	-
- Commercial	6.78	2.45–9.45	0.135	-	-	-
**700 m Distance (153.9 Hectares)**						
Parameters	OR	95% CI	*p*	OR	95% CI	*p*
Demographic and clinical factors:						
Sex	1.01	0.39–2.64	0.979	-	-	-
Age	0.97	0.92–1.02	0.203	-	-	-
BMI	1.02	0.99–1.05	0.276	-	-	-
HA	1.75	0.69–4.42	0.236	-	-	-
DM	1.34	0.46–3.90	0.592	-	-	-
Hyperlipidemia	2.25	0.89–5.70	0.087	4.24	1.34–13.39	0.014
CV disease	1.65	0.50–5.47	0.409	-	-	-
Coronary angiography:						
Primary Gensini	1.01	0.99–1.03	0.392	1.02	1.0–1.05	0.112
Primary PCI	1.71	0.62–5.03	0.286	-	-	-
Time interval	1.00	0.99–1.00	0.611	-	-	-
Development:						
- Housing	0.59	0.02–1.23	0.747	0.03	0.00–0.49	0.025
- Industrial	2.56	1.67–4.87	0.535	-	-	-
- Commercial	12.67	2.45–16.56	0.113	-	-	-

Abbreviations: BMI—body mass index, CI—confidence interval, COPD—chronic obstructive pulmonary disease, CV—cardiovascular, HA—arterial hypertension, DM—diabetes mellitus, OR—odds ratio, PCI—percutaneous intervention.

## Data Availability

Data will be made available after reasonable request by contacting the corresponding author.
